# Efficiency of Ferritin bio-nanomaterial in reducing the pollutants level of water in the underground corridors of metro rail using GIS

**DOI:** 10.1038/s41598-022-24626-3

**Published:** 2022-11-24

**Authors:** R. Lilly, S. Prabhakaran, K. Giridharan, Padmanabhan Sambandam, B. Stalin, S. J. Subhashini, N. Nagaprasad, Leta Tesfaye Jule, Krishnaraj Ramaswamy

**Affiliations:** 1 Department of Naval Architecture and Offshore Engineering, Academy of Maritime Education and Training, Chennai, Tamil Nadu 603112 India; 2 Department of Marine Engineering, Academy of Maritime Education and Training, Chennai, Tamil Nadu 603112 India; 3 Department of Mechanical Engineering, Easwari Engineering College, Chennai, Tamil Nadu 600089 India; 4 School of Mechanical and Construction, Vel Tech Rangarajan Dr.Sagunthala R&D Institute of Science and Technology, Chennai, Tamil Nadu 600062 India; 5 Department of Mechanical Engineering, Anna University, Regional Campus Madurai, Madurai, Tamil Nadu 625 019 India; 6 Department of Computer Science and Engineering, School of Computing, Kalasalingam Academy of Research and Education (Deemed to be University), Virdhunagar, Tamil Nadu 626126 India; 7 Department of Mechanical Engineering, ULTRA College of Engineering and Technology, Madurai, Tamil Nadu 625 104 India; 8 Centre for Excellence-Indigenous Knowledge, Innovative Technology Transfer and Entrepreneurship, Dambi Dollo University, Dembi Dolo, Ethiopia; 9 Department of Physics, College of Natural and Computational Science, Dambi Dollo University, Dembi Dolo, Ethiopia; 10 Department of Mechanical Engineering, Dambi Dollo University, Dembi Dolo, Ethiopia

**Keywords:** Environmental sciences, Engineering, Materials science, Nanoscience and technology

## Abstract

The underground developments are likely to deteriorate the water quality, which causes damage to the structure. The pollutant levels largely affect the aquifer properties and alter the characteristics of the water quality. Ferritin nanoparticle usage proves to be an effective technology for reducing the pollutant level of the salts, which are likely to affect the underground structure. The observation wells are selected around the underground Metro Rail Corridor, and the secondary observation wells are selected around the corridors. Ferritin is a common iron storage protein as a powder used in the selected wells identified in the path of underground metro rail corridors. Water sampling was done to assess the water quality in the laboratory. The water quality index plots for the two phases (1995–2008) and (2009–2014) using GIS explains the water quality scenario before and after the Ferritin treatment. The Ferritin treatment in water was very effective in reducing the pollutants level of Fluoride and sulphate salts which is likely to bring damage to the structure.

## Introduction

The water quality status due to the construction of the underground metro rail corridor has greatly altered due to the changes in the aquifer properties, and the heterogeneous layer broke down below the surface of the ground.

### Ferritin

The protein that contains iron is generally called Ferritin, and it regulates mineralization. Ferritin is a storage protein that may be found in both mammals and plants. Ferritin is made up of a cage-like protein structure made up of 24 structurally identical polypeptides^1^. Solar radiation plays a major role in the protein shell, which stops the mineralization process. Ferritin has been revealed to be capable of remediating harmful metals and chlorocarbons^2^. The following are some of the benefits of ferritin as a catalyst above regular iron: Ferritin is more stable and does not respond under photoreduction. The conversion of chromium Cr (VI) to Cr (III) is one obvious application of ferritin that has been established in lab^3^. Cr (VI) is a carcinogenic contaminant found in industrial waste, whereas Cr (III) is generated naturally as a Cr compound and is less dangerous and water-insoluble^4^.

### Ground water quality and management

The quality of groundwater is determined by all of the processes and reactions that occur between the time it condenses in the atmosphere and the time it is released by a well. As a result, the quality of groundwater varies with location, water table depth, and season and is mostly determined by the dissolved solids present. The substructure development impacts the groundwater quality and has adverse effects on it. The various types of sub-structures are underground drainage, water lines, and depth not more than 4 m, but the sub-structure development, like tunneling, breaks and changes the geological formations, water level, and water quality.

The efficient management of existing groundwater resources as a source of water supply to meet current needs on a long-term basis in an equitable manner while maintaining its quality, without negotiating the risks associated with damage to aquifer physical characteristics, storage capacity, and recovering ability for future generation needs, is referred to as sustainable groundwater resource development. Knowledge of the behavior of groundwater systems and their interactions with the environment is critical for developing effective, long-term management strategies^5^. Excessive pumping and rising demand in some Indian cities are putting millions of people at risk of running out of groundwater reserves. Climate variability could be a crucial factor influencing the rainfall and groundwater recharge relationship, according to statistical correlation research^6^. Urbanization causes predominant changes in the water quality pattern, and it seems to be a highly affecting parameter in any area considered for the study. Sampling has to be done for a large number of wells for the accurate prediction of the changes in the water quality pattern in study area^7^. Due to increasing industrialization and urbanization, the environment is facing various threats like river pollution, soil degradation, and polluted air quality^8^. Quality indices appear to be very useful in evaluating these conditions. The aquifer properties below the ground surface and the type of aquifer present below the surface of the ground are likely to be the predominant reason for the changes occurring in the water quality^9^.

### GIS approach

The GIS approach is utilized in numerous researches to examine the influence of urbanization on groundwater quantity and quality^10^. Statistical weighting schemes and simple overlay analysis themes in GIS are used to study the impact of urbanization in terms of Water Quality^10–12^. It clearly says that data collection is the basic work of GIS construction^13^. It is evident that the integrated Geographical Information System has wide applications in environmental studies, geoscientific approaches, and urban utilities^14^. Water quality index determination is a widely accepted methodology to rate water quality. Remotely-sensed data, Existing maps, and Field data are used in the GIS study. Various steps like conversion of data to digital format, generation of thematic layers, and generation of the GIS database followed by interpolation and analysis are the general procedures involved in GIS^12^.

### Water quality index

WQI is interpreted as a measure that reflects the combined effect of many water quality criteria^15^. The water quality index (WQI) is the most widely used indicator for categorizing and providing a general view of water quality standards by converting water quality information received from numerous criteria into a single value/rating. The use of a geographical information system (GIS) to analyze and map groundwater quality has proven to be useful in detecting areas where groundwater quality is worsening. In the GIS environment, interpolation techniques such as inverse distance weighting (IDW) and kriging were used to measure the geographic variability of groundwater quality^16^. Water Quality Index is a powerful methodology to determine the water quality status in an area. The spatial variations can be clearly shown in a map using vector analysis in GIS^10^.

### Characteristics of nanoparticles

Ferritin nanoparticles, when it gets exposed to the atmosphere, the photocatalyst reaction tends to convert the protein into radical when the iron content is high and superoxides when it is used in the lower level, which is very dangerous to the water environment^17^. The light in the environment reacts with the chemicals of ferritin, and the UV rays are subjected to a photocatalyst reaction and convert the chemicals into peroxides^18^. Nanomaterials have different unique characteristics. It improves the optical and magnetic properties^19^. Nano photocatalysts generate oxidizing species and increase their oxidation ability. It effectively aids in the breakdown of contaminants from polluted water^20^.

Nanoparticles such as zero-valence metals, semiconductors, and some bimetallic types are commonly employed to address environmental pollutants such as azo dyes, Chlorpyrifos, organochlorine insecticides, nitroaromatics, and others^21–24^. TiO_2-_based nanotubes can effectively remove pollutants from wastewater^24,25^. Organic pollutants such as azo dyes and chlorinated ethane are effectively reduced using nanoparticles^26–28^. SiO_2_, ZnO, TiO_2_, Al_2_O_3_, and other metal oxide nano photocatalysts are the most prevalent and significant^29–31^.

Titanium dioxide (TiO_2_) is a photocatalyst that seems to be the best one in terms of low cost and effectiveness in removing the levels of the toxic pollutant. Its easy availability is found to be the versatile nature of the chemical. Until now, anatase has been thought of as a promising nano photocatalyst^32,33^. 3.2 eV bandgap and is capable of absorbing < 387 nm UV rays^34,35^. ZnO, another photocatalyst, is used to remove the pollutants effectively from wastewater^36,37^.

Escherichia coli is found to be the source of wastewater, and it seems to be very toxic in nature. Pd included ZnO nanomaterial is used to remove this pollutant from wastewater due to effective photocatalytic reactivity^38^. The performance of the photocatalytic under radiation in the presence of visible light brings a new focus toward metal oxides with the modification in metal ions^35,39^. Many carbonaceous materials need a modification in the nano photocatalyst and also for the dye sensitizers^40^. SPA method using interval-based fuzzy numbers (IFN) has become popular in water quality assessment^41^.

Hence, the objective of the paper is to examine the pollutant level concentration after the usage of ferritin nanoparticles and represent the water quality status using the GIS environment. The water quality scenario was evaluated using the water quality index, which compares temporal fluctuation in the Metro rail Environment of the observation wells. Water quality index determination was used to assess the state of the water quality in the research region.

## Materials and methods

### Ferritin

**Figure 1:**
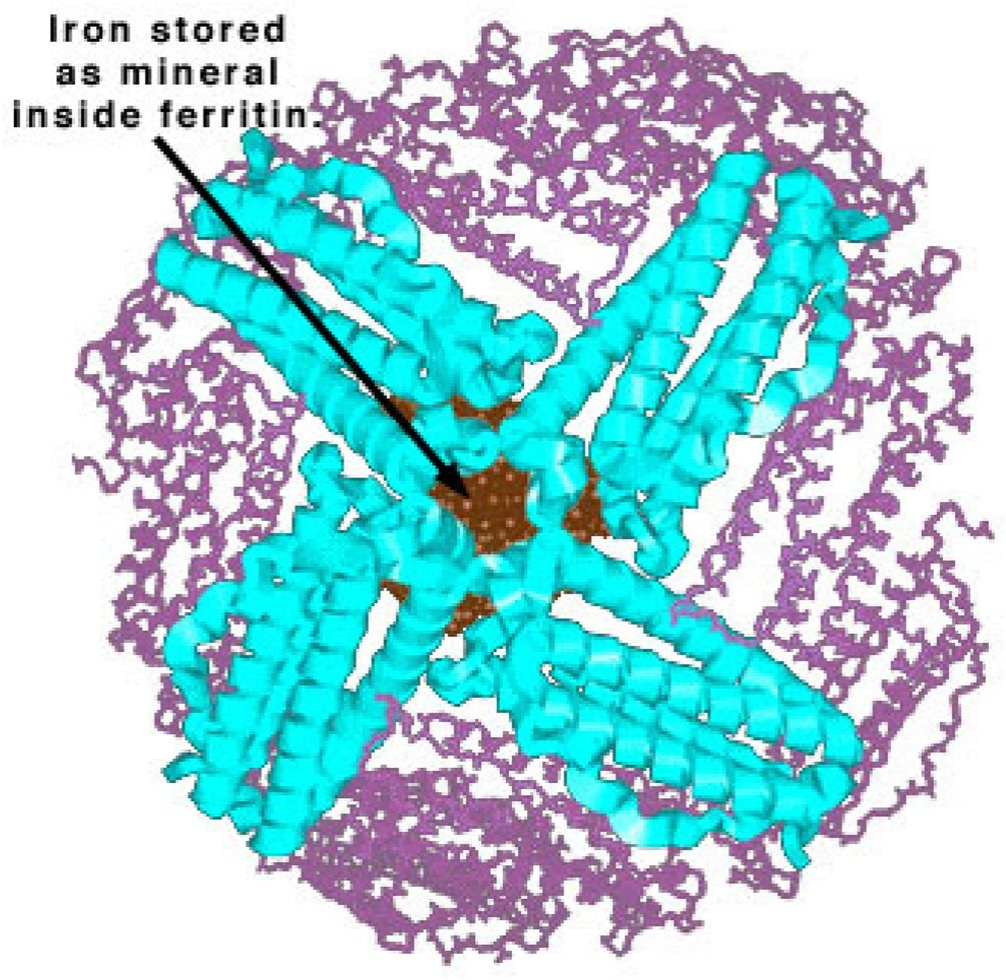
Ferritin structure. (Source**:** chemistry.wustl.edu).

Ferritin storage protein is highly soluble, and its size is less than 10 nm. It consists of 24 subunits, each with a molecular weight of about 20,000, and its density is 2.5 times that of apoferritin. It comprises molecules with varied iron content that does not exceed 3000. The storage protein is colorless and sharp. Its boundary consists of iron-free protein. The ferritin structure is shown in Fig. 1.

Ferritin has unique properties both chemically and physically. It is able to withstand temperatures up to 75 °C, and it is very stable for various denaturants. It has the ability to break down at a pH of 2.5 in an acidic environment and gets restored to a pH of 7.5. This unique finds to be very effective in water purification and also maintains the pH. Ferritin storage protein deals with environmental pollutants in two ways. The exchange of electrons and the production of oxygen are the two processes that involve water pollutant reduction. When excess irons are exposed to the atmosphere, they react and generate radicals which in turn destroy the protein. When it is used at a lower level, the cell generates superoxide, which also stops the affinity of the pollutants to the protein. Hence the correct proportion of ferritin has to be utilized for the given environment.

Ferritin protein is of size less than 10 nm, and it is available in powder form when it is used in an open environment. The wind is the only factor that is likely to affect the spreading of ferritin protein into the water evenly. Hence while using the storage protein, some closed arrangement has to be done to use it effectively. Pyrococcus furiosus ferritin is the source of ferritin-NP, which has ferric phosphate as its main component. The ferritin from Pyrococcus furiosus is structurally comparable to ferritins from other bacteria and eukaryotes. It's a 24-mer with 20 kDa subunits totaling 480 kDa in size. Pyrococcus furiosus ferritin protein belongs to the type of Non-heme ferritin. The ferritin from Pyrococcus furiosus is particularly thermostable. The half-life of ferritin activity is 48 h at 100 °C and 85 min at 120 °C. There was no apparent melting temperature up to 120 °C. The exceptional thermostability of Pyrococcus furiosus ferritin could be beneficial in biotechnology.

Inside cells, ferritin is a 24-mer iron (Fe) storing protein. The molecular weight was higher than expected at 474 kDa, and the hydrodynamic size was 18 nm. It was discovered that there was a negative charge present. The quality of a nanoparticle is influenced by particle size, shape, solid-state characteristics, and physical stability. Other characteristics that influence nanoparticle quality include further chemical structural degradation, dissolving, and solubility testing. The study employed ferritin-NP particles with a diameter of 1.7 nm to 0.9 nm and an irregular pyramidal form with a base side of 150 m and a height of 250 m. Iron is the most important element in the nanoparticle, and it is extremely thermally stable in nature.

### Study area

**Figure 2:**
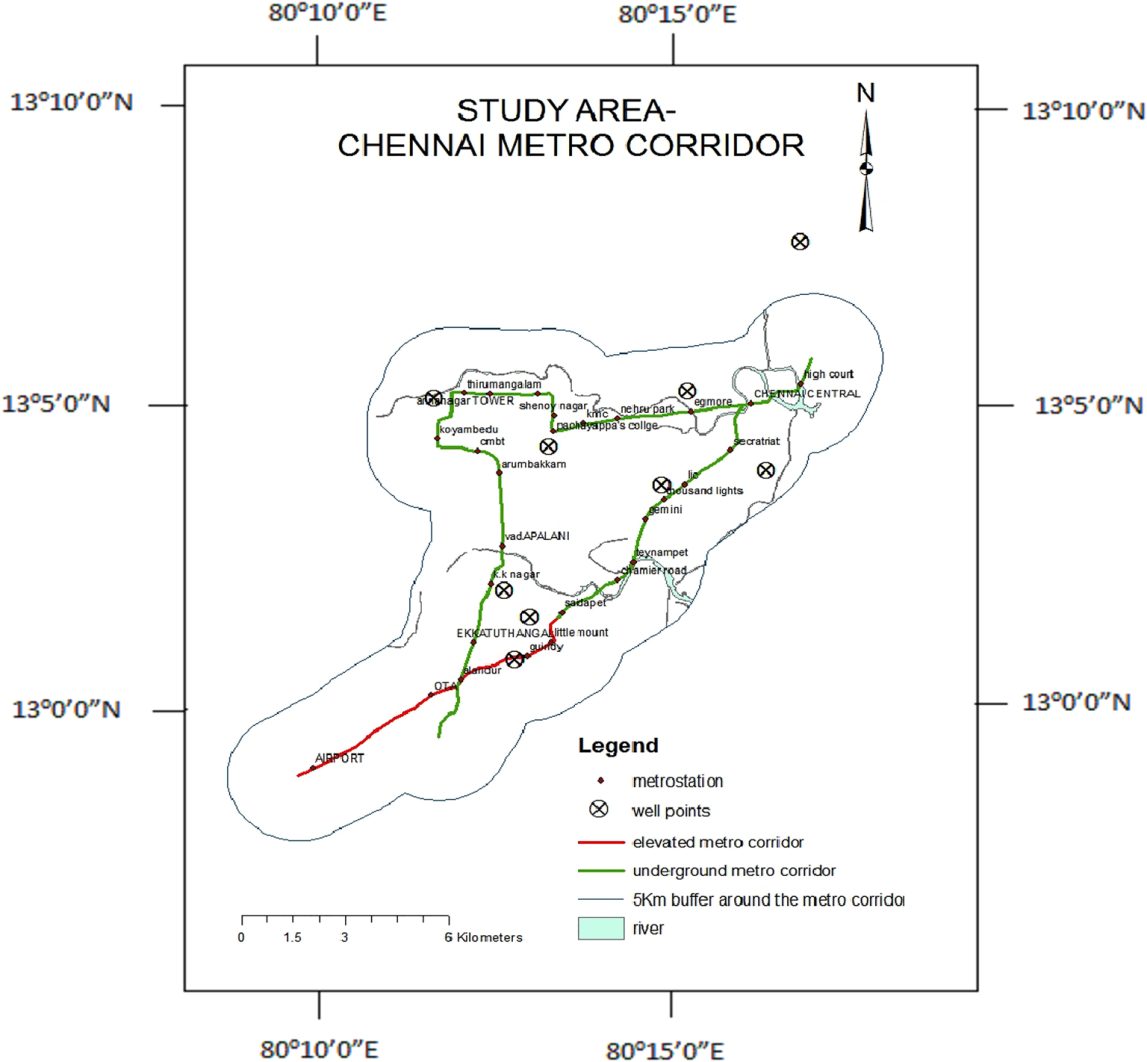
The observation wells in the study area of the underground Metro Rail Corridor, Chennai, Tamilnadu, India.

Chennai is a well-known and attractive city due to its urbanization. Today it has attained the status of being called a Metro City in India. It is located with a latitude of 13.0827° N, and a longitude of 80.2707° E. It covers a geographical area of about 174 Sq.km with a population of around 68 lakhs and an average annual rainfall of 1200 mm. The observation wells were selected from the available data, and these were close to the Metro Rail Corridors. The observation wells in the study area are shown in Fig. 2.

The map consists of ten observation wells selected in and around the Metro Rail Corridors and the Metro stations. The elevated and the underground Metro Rail Corridors have been differentiated with different colors. The Metro Rail Corridors are buffered for a distance of about 5 km, and the Adyar and Coovam Rivers are located.

The observation wells are selected to be on the two sides of the underground corridors to study the impact of the construction. The aquifer damage and the breakdown of the heterogeneous layers are considered to be the predominant factors that might cause the changes in the water quality. Hence the wells which are identified should be in the vicinity enough to have the effect of ferritin protein spread in the water.

### Climate

Chennai is in the south of the nation, where it is frequently hot and muggy. The three main seasons in Chennai are summer, monsoon, and winter. The summer season in Chennai is seen as lasting from March to June. The NE monsoon period is the time between October and December. The months of June through September make up the monsoon season. The brief winter season in Chennai lasts from November to February.

### Rainfall

**Figure 3:**
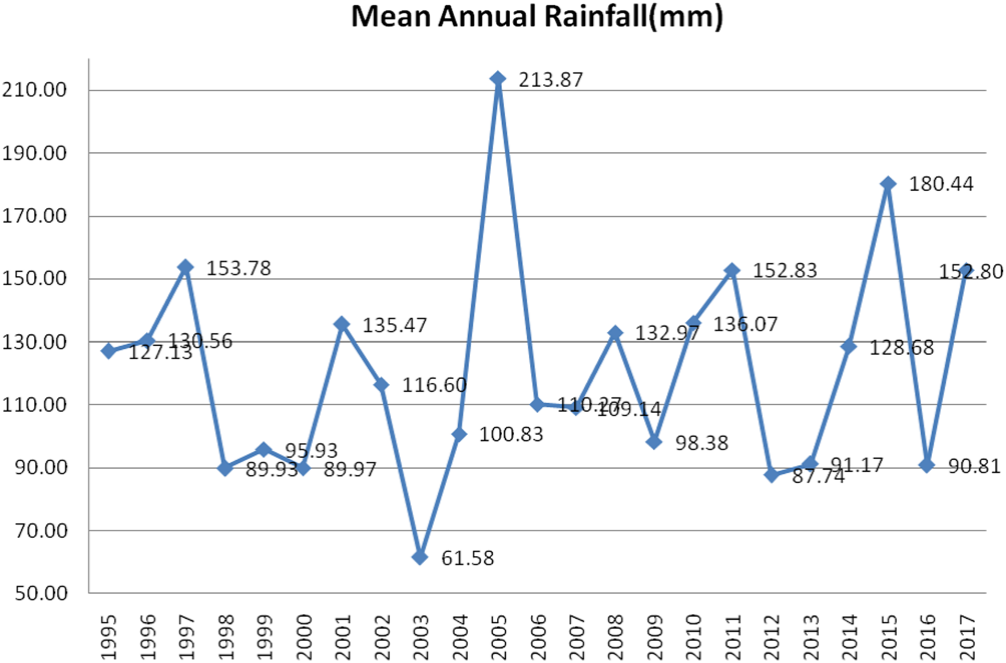
Yearly average rainfall in the study area (1995–2017).

Nearly 65% of the rain falls during this season; the NE monsoon is a major factor in Chennai's weather. An annual average of 1,300 mm of moderate rainfall falls in Chennai city. Figure 3 displays the annual average rainfall from 1995 to 2017. In the city, 213.87 mm of rainfall per year on average was observed in 2005. Due to the enormous storm that hit Chennai recently, the city experienced its highest average annual rainfall in 2015.

### Temperature

The summer starts around the end of March and continues till June, with late May to June being the hottest months. Temperatures frequently get above 40 degrees Celsius during this time. The climate and weather in Chennai are rather stable, with little seasonal temperature variance, because of the city's closeness to the sea and the thermal equator. The city typically faces heat in the summer months, with average maximum temperatures ranging from 38 to 42 degrees Celsius. The city experiences a brief winter, with January being the coolest month, with lows of 18 to 20 degrees Celsius.

### Geology

**Figure 4:**
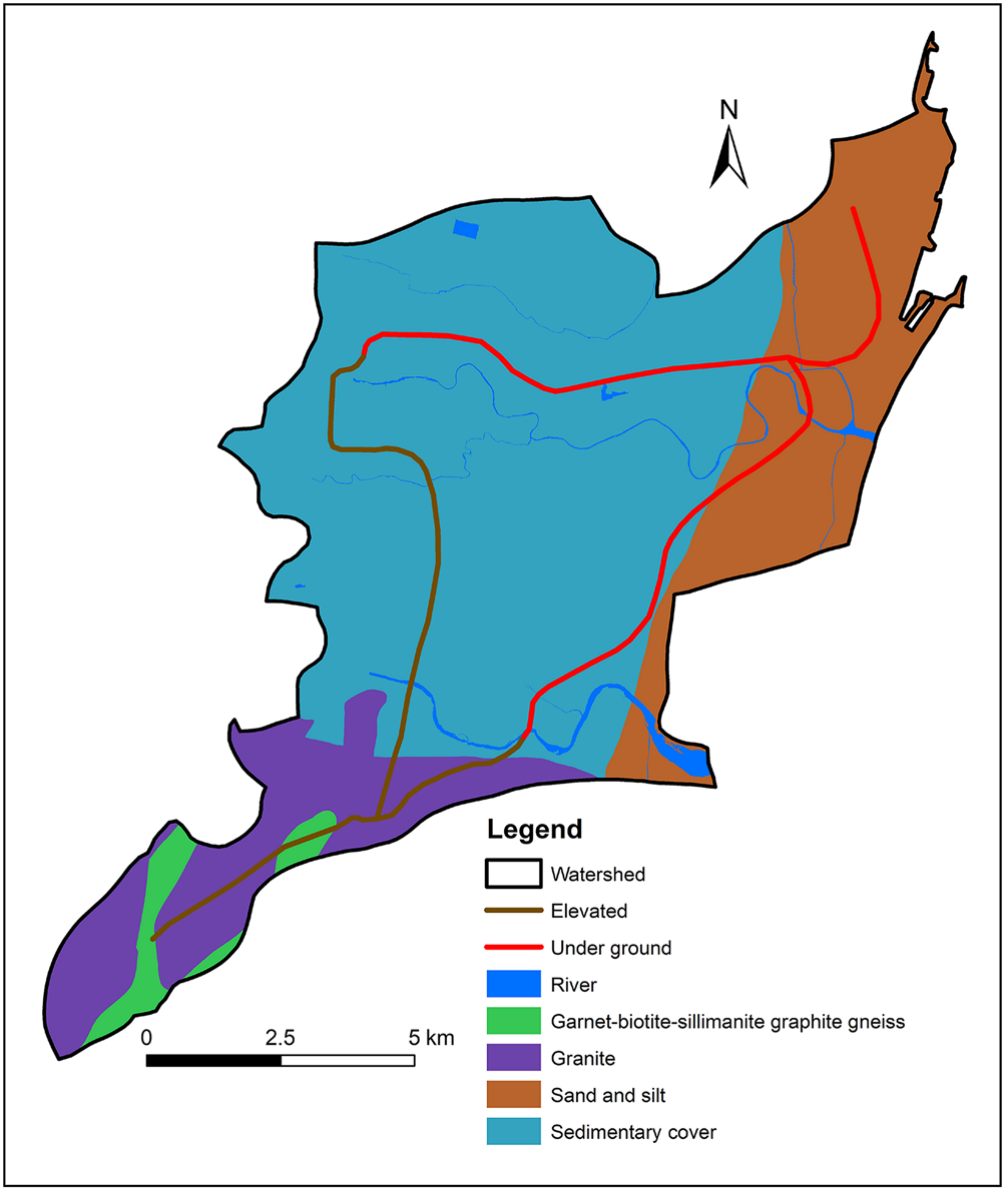
Geological classifications of Study area.

The Geological Survey of India, Chennai, provided a map of the research region at a scale of 1:50,000, which was used for digitizing the area's geology. Figure 4 depicts the various geological formations of the research region. The study area comprises marine beds of Neocomian age and the earliest marine transgression layers of the middle Cretaceous of Upper Albianage, as well as coastal sediments on the bank side and a portion of archean rocks composed of Chamockites, Granite, and Gneisses. The second subterranean section runs from Thirumangalam to Egmore Metro and is made up of newer alluvium with thicknesses ranging from 3 to 30 m and tertiary rock from the Eocene to Pliocene eras that contain the composition of sandstone.

### Ferrite treatment using a reaction chamber

Magnetic seeding is an efficient process that makes magnetic suspended particles bind by themselves, and the pollutants are removed under various procedures. Various methods like coagulation and ferromagnetic suspension are done for particles of very fine sizes. The ferritin-NP is used at the rate of 0.3 × 10^–3^ kg/m^3^ in the polluted water of the underground corridor. The required amount of ferritin –NP needed for the investigation was procured in the research conducted in the chemical department AC-Tech of Anna University. The particles which are finer than the silt are removed through adhesion, magnetic separation, and absorption techniques. These techniques are considered to be the primary phase in the ferrite treatment procedure. The investigations involved in the treatment of water below the underground corridors with the ferrite solution are also conducted.

Aggregate distribution modeling is the challenging part before it gets into the mechanical mixer. In the reaction chamber, the magnetic aggregates and the ferrite solution water has to mix in the proper volume ratio so that it meets the uniform distribution of the aggregates in the reaction chamber. The process of gravity and diffusion turbulence is used to observe the magnetic aggregates distribution, and the vertical transfer regularities in the presence of light were investigated. Inertial force is found to be modest when compared to hydrodynamic force. It ensures uniform and regular distribution across the diameter of the mixer for the mixer height. The following modeling equation expresses fluxes in the particle arrangement and their movement in the opposite direction. It is represented as.

1 $$Dt\frac{dC}{{dh}} + WagC = 0$$

D_t_, Coefficient of turbulent diffusion [m^2^/s]; C, Concentration of the particle at height h [kg/m^3^]; W_a_, Aggregates settling velocity [m/s].

By integrating Eq. (1) with boundary conditions *C* = Co, *h* = const,

We obtain

$$C = Co\exp \left( {\frac{Wagh}{{Dt}}} \right)$$

The water-soluble ferritin powder is used in the selected wells around the Metro Rail Corridor. The dosage will be determined according to the water level of the wells. Using the area and the height of the water level, the volume is determined. According to the volume ratio the required dosage of the ferritin is determined, and it is added to the wells. Leaving the well for a period of one week, the effect of ferritin in reducing the pollutant level was analyzed by properly locating the borehole points around the Metro Rail Corridor.

The sampling points are located around the foundation of the structure, and the water sampling is done based on Indian standards. The sampling water was taken to the laboratory, and the pollutant levels were analyzed.

### Determination of water quality parameters

The various water quality parameters are determined in the laboratory by the following methods. The well water was collected either by pumping or by hand, and samples were taken from the well bottoms in clean polyethylene bottles, carried in iceboxes, and refrigerated until assessment at 4 °C. Quality assurance and quality control was assured by doing repeated calibrations, and accuracy was obtained both in devices and also in the titration methods. Separate samples were obtained for Physico-chemical analysis. The study's physical parameters included pH, Total Dissolved Solids (TDS), and Total Hardness (TH). The chemical parameters included anions such as Fluoride (Fl) and Chloride (Cl). The various detection methods are given in Table 1.

**Table 1:** Laboratory test used for the detection of various pollutants.

S.No	Pollutant	Detection method
1	pH	pH meter
2	TDS or Electrical conductivity	TDS or Conductivity meter
3	Total hardness	Titration using dye indicators
4	Calcium, Magnesium	Argentometric method
5	Fluoride	Ion selective Electrode method
6	Sulphate	Spectrophotometric method

Centre Ground Water Board and Institute of Water Studies, Chennai, are the data sources used to collect the water quality data for the concerned observation wells around the underground Metro Rail Corridor. The period from 1995–2014 was used for the data collection. The construction of Chennai Metro Rail was started in the year 2008; hence two periods of data such as (1995–2008) and (2009–2014) that are is before and after the construction, were taken for the determination of the values of pH, EC, TDS, TH, Ca, Mg, TA, Cl, F, and SO_4_. The Mean values were tabulated, and the comparison is shown in Table 2.

**Table 2:** Water quality parameters comparison for the two phases.

Place	Latitude	Longitude	pH		EC (µs/cm)		TDS (mg/l)		TH (mg/l)		Ca (mg/l)	
			1995–2008	2009–2014	1995–2008	2009–2014	1995–2008	2009–2014	1995–2008	2009–2014	1995–2008	2009–2014
Tandiarpet	13° 07′ 38″ N	80° 17′ 24″ E	8.2	7.4	1937.22	1239.33	1162.67	1871.00	406.39	282.50	52.78	58.00
Vepery	13° 05′ 07″ N	80° 15′ 38″ E	8.0	7.7	4167.00	3952.25	2327.20	2900.00	722.00	662.50	76.88	57.00
Chepauk	13° 03′ 48″ N	80° 16′ 52″ E	8.0	7.8	1767.33	2236.57	1002.07	1432.00	333.33	356.07	73.07	71.43
ThousandLights	13° 03′ 32″ N	80° 15′ 05″ E	7.9	7.9	2471.88	2471.88	1032.07	1445.00	475.14	475.14	123.43	123.43
Saidapet	13° 01′ 20″ N	80° 13′ 10″ E	8.0	8.2	919.41	943.33	564.26	935.00	281.47	232.08	55.53	51.00
Guindy	13° 0′ 37″ N	80° 12′ 56″ E	8.0	7.6	919.41	1485.75	564.26	980.00	281.47	200.00	55.53	31.00
Aminjikarai	13° 04′ 12″ N	80° 13′ 28″ E	8.4	7.4	1294.44	1762.75	782.27	982.00	243.89	476.25	31.00	99.00
Tirumangalam	13° 05′ 00″ N	80° 11′ 40″ E	7.8	7.4	2288.30	1884.40	752.17	1193.00	472.00	345.00	105.78	49.20
Vadapalani	13° 03′ 16″ N	80° 12′ 41″ E	8.4	8.2	1294.44	1290.00	862.07	1186.80	243.89	235.00	31.00	29.00
K.K.Nagar	13° 01′ 47″ N	80° 12′ 47″ E	7.8	7.6	1517.27	1219.50	642.17	1127.00	387.00	220.00	90.40	48.50
Airport	12° 59′ 38″ N	80° 10′ 19″ E	7.5	7.8	900.30	1320.00	565.30	753.00	285.30	285.30	54.20	54.20
Mean Value			7.99	7.72	1770.64	1800.52	932.41	1345.89	375.63	342.71	68.14	61.07

Place	Latitude	Longitude	Mg (mg/l)		TA (mg/l)		Cl (mg/l)		F (mg/l)		SO4 (mg/l)	
			1995–2008	2009–2014	1995–2008	2009–2014	1995–2008	2009–2014	1995–2008	2009–2014	1995–2008	2009–2014
Tondiarpet	13° 07′ 38″ N	80° 17′ 24″ E	66.8	34.9	275.00	75.00	372.94	178.89	0.00	0.87	161.39	118.88
Vepery	13° 05′ 07″ N	80° 15′ 38″ E	128.9	124.1	175.00	120.00	823.70	703.76	0.56	1.16	401.44	680.71
Chepauk	13° 03′ 48″ N	80° 16′ 52″ E	36.4	42.7	175.00	135.00	337.60	494.63	0.34	0.57	144.67	137.36
ThousandLights	13° 03^′^ 32″ N	80°15′05″ E	40.6	40.6	150.00	150.00	555.14	555.14	0.62	0.62	118.80	118.80
Saidapet	13° 01′ 20″ N	80° 13′ 10″ E	34.8	24.9	175.00	225.00	113.18	110.67	0.38	0.32	86.56	43.00
Guindy	13° 0′ 37″ N	80° 12′ 56″ E	34.8	28.3	175.00	100.00	113.18	187.01	0.38	0.52	86.56	68.21
Aminjikarai	13° 04′ 12″ N	80° 13′ 28″ E	40.3	66.8	300.00	75.00	258.00	392.70	0.34	0.49	99.61	85.50
Tirumangalam	13° 05′ 00″ N	80° 11′ 40″ E	57.0	55.4	125.00	75.00	523.80	428.98	0.80	0.50	114.63	131.34
Vadapalani	13° 03′ 16″ N	80°12′ 41″ E	40.3	42.0	300.00	225.00	258.00	270.00	0.34	0.25	99.61	110.00
K.K.Nagar	13° 01′ 47″ N	80° 12′ 47″ E	39.2	24.0	125.00	100.00	265.50	161.43	0.76	0.72	87.38	92.89
Airport	12° 59′ 38″ N	80° 10′ 19″ E	32.6	32.6	85.00	135.00	100.30	110.00	0.30	0.72	90.30	90.30
Mean Value			50.15	46.93	187.27	128.64	338.30	326.66	0.48	0.61	135.54	152.45

**Figure 5:**
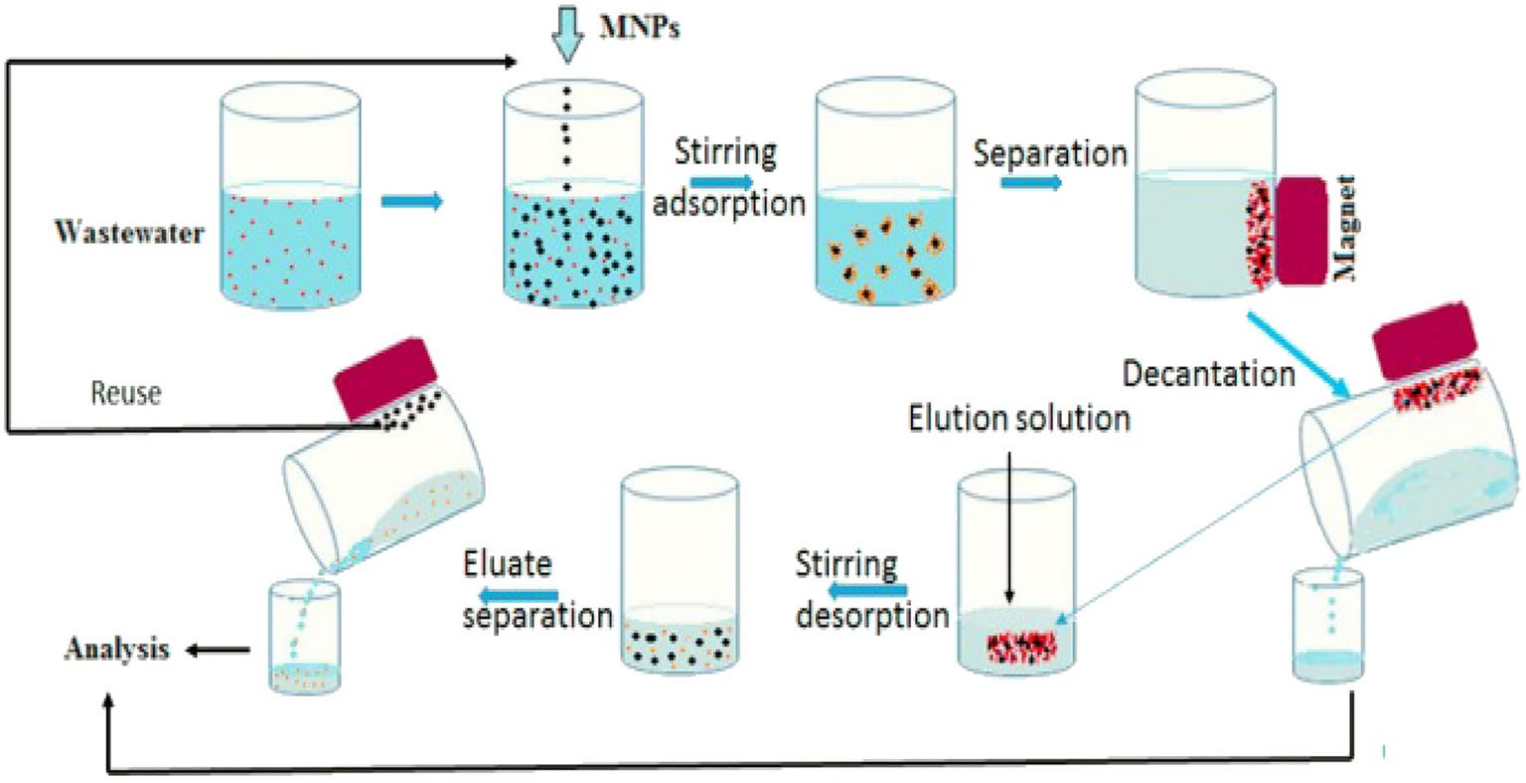
The process of separation of pollutants in the reaction chamber.

The values of pH, Total alkalinity, calcium, and chloride were decreased in the 2009–2014 phase when compared to 1995–2008. The decrease in rainfall and the underground tunneling reduced runoff, and hence the suspended form of organic and inorganic substances, i.e., Total dissolved solids got, increased. Since electrical conductivity and total dissolved solids are directly related to each other, it also showed an increase in pattern. Bentonite slurry, which consists of minerals like Potassium, Sodium, Calcium, and Aluminium was issued for tunneling work, confirming the presence of calcium. The sulphate concentration showed an increase in nature; it may be due to the use of gypsum (CaSo_4_) in the binder during construction. The natural weathering of the rocks induces the fluoride concentration in an abundant manner. Tunneling invokes artificial weathering by breaking down the rocks, and this may be a cause of the increase in the fluoride content in the later phase. The processes of separation of the pollutants are given in Fig. 5.

The water pollutants in the samples of the underground Metro Rail Corridor were collected according to the standard sampling procedure. It was subjected to the reaction chamber. In the reaction chamber, the ferritin petals are added to the wastewater volume ratio. It’s been initially subjected to an adsorption process where the cohesive particles are stuck to the surface of the chamber. This process is still activated by stirring. After the adsorption process, the liquid is subjected to a separation process. The decanted residue was subjected to a desorption process where the pollutants are dissipated, followed by a separation process which will give the pollutant level reduced water.

**Figure 6:**
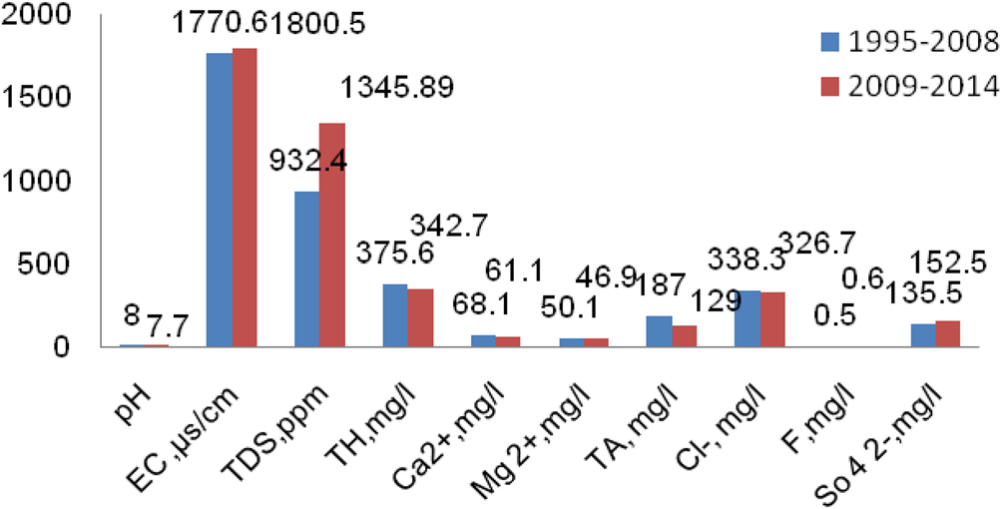
Comparison between the mean value parameters.

Figure 6 clearly shows the water quality parameters comparison before the ferritin treatment and after the ferritin treatment process in the observation wells around the underground Metro Rail Corridor.

### Efficiency achieved by the ferritin in the removal of pollutant levels

The volume of the water allowed in the reaction chamber is at the rate of 1–9 m^3^/h. The dosage of ferritin was determined according to the volume of the wastewater ratio. The pollutant levels of the water collected from the Metro Rail Corridor was observed from the various laboratory test, and the mean values of the levels are noted for the phase from 1995 to 2008.

The contaminants of water, when subjected to ferritin petals, react with water and disintegrate the pollutants, the adsorption process makes the pollutants get dispersed and it is attached to the surface. Hence the pollutant levels, which are at their worse, deteriorate, the foundation of the structure will be get reduced, and the life of the structure will be increased. Basically, ferritin is an iron storage protein that absorbs alkali salts and reduces the pH value. The electrical conductivity and the Total Dissolved solids will be increased only in the presence of salts. Once the ferritin nanoparticles absorb the salts, the electrical conductivity and total hardness are reduced. The water-soluble protein reduces excess fluoride and sulphate salts. Hence the hardness of the water is also very much decreased.

As ferritin protein nanoparticles are of size less than 10 nm. It is available in the finest powder form. The concentration of the particle weight has to be carefully mixed with water for better results. In the open environment, the wind is the one predominant factor that reduces the effectiveness of mixing with the water. Hence care should be taken to mix up the chemicals in the closed environment for the précised outcome.

## Results and discussion

### Water quality index determination

The selected parameters were assigned a weight relative to their importance in drinking water quality. Parameters such as pH, EC, TDS, F, and So_4_ were given much importance and assigned the value 4. Total Hardness, Total Alkalinity, and chloride were assigned 3. Calcium and magnesium carried 2.

Utilizing the method of the weighted arithmetic index, the relative weight (Wi) was computed in the following procedure:

2 $${\text{W}}_{{\text{i}}} = {\text{w}}_{{\text{i}}} /\Sigma {\text{w}}_{{\text{i}}}$$

where W_i_ is the relative weight, n is the number of parameters, and w_i_ is the weight of each parameter.

The concentration of each water sample (mg/l)

3 $${\text{Quality}}\,{\text{rating}}\;{\text{Scale}}\,({\text{Q}}_{{\text{i}}} )$$

Respective standard as per guidelines BIS 10500.

The Sub Index (SI_i_) is calculated using the formula;

4 $${\text{SI}}_{{\text{i}}} = {\text{W}}_{{\text{i}}} \times {\text{Q}}_{{\text{i}}}$$

for each parameter. The overall WQI is calculated by adding all the sub-index values of each groundwater sample as follows:

5 $${\text{WQI}} = \Sigma\; {\text{SI}}_{{\text{i}}}$$

The classification of water quality based on the water quality index value and its status is shown in Table 3.

**Table 3:** Classification of water quality based on WQI value.

Class	Value of WQI	Status of water quality
I	< 50	Excellent
II	50–100	Good Water
III	100–200	Poor Water
IV	200–300	Very poor Water
V	> 300	Water unsuitable for Drinking

Water quality index values determined from Tables 4 and 5 for the two periods clearly show that water quality after construction deteriorated. The variation between the water quality index values is very less. Hence it is observed that due to the development of the sub-structure, water quality status almost remains the same.

**Table 4:** WQI determination for 1995–2008.

S.No	Parameter	Indian standards (2005)	Mean value	Min value	Max value	Weightage (w_i_)	Relative weight (W_i_)	Quantity rating (q_i_)	Sub index (SI_i_)
1	pH	6.5–8.5	8.0	7.5	8.4	4	0.1212	94.11	11.406
2	EC, μs/cm	500–2000	1770.6	900.3	4167	4	0.1212	88.53	10.729
3	TDS, ppm	500–2000	932.4	564.3	2327.2	4	0.1212	46.62	5.650
4	TH, mg/l	300–600	375.6	243.9	722	3	0.0909	62.6	5.690
5	Ca^2+^, mg/l	75–200	68.1	31	123.4	2	0.0606	34.05	3.036
6	Mg^2+^, mg/l	30–100	50.1	32.6	128.9	2	0.0606	50.1	3.036
7	TA, mg/l	200–600	187	85	300	3	0.0909	31.17	2.833
8	Cl^-^, mg/l	250–1000	338.3	100.3	823.7	3	0.0909	33.83	3.075
9	F, mg/l	1–1.5	0.5	0.3	0.8	4	0.1212	33.33	4.039
10	So_4_^2−^, mg/l	200–400	135.5	86.6	401.4	4	0.1212	33.875	4.106
						Σ w_i_ = 33			Σ SI_i_ = 52.63

**Table 5:** Water quality index determination for 2009–2014.

S.No	Parameter	Indian standards (2005)	Mean value	Min value	Max value	Weightage (w_i_)	Relative weight (W _i_)	Quantity rating (q _i_ )	Subindex (SI _i_)
1	pH	6.5–8.5	7.7	7.4	8.2	4	0.1212	90.58	10.978
2	EC, μs/cm	500–2000	1800.5	943.3	3952.3	4	0.1212	90.025	10.911
3	TDS, ppm	500–2000	1345.89	753	2900	4	0.1212	67.29	8.155
4	TH, mg/l	300–600	342.7	200	662.5	3	0.0909	57.12	5.192
5	Ca^2+^, mg/l	75–200	61.1	29	123.4	2	0.0606	30.55	1.851
6	Mg^2+^, mg/l	30–100	46.9	24	124.1	2	0.0606	46.9	2.842
7	TA, mg/l	200–600	120	75	225	3	0.0909	21.5	1.954
8	Cl^−^, mg/l	250–1000	326.7	110	555.1	3	0.0909	32.67	2.969
9	F, mg/l	1–1.5	0.6	0.3	0.72	4	0.1212	40	4.848
10	So_4_^2−^, mg/l	200–400	152.5	43	680.7	4	0.1212	38.125	4.621
						Σ w_i_ = 33			Σ SI_i_ = 54.321

**Figure 7:**
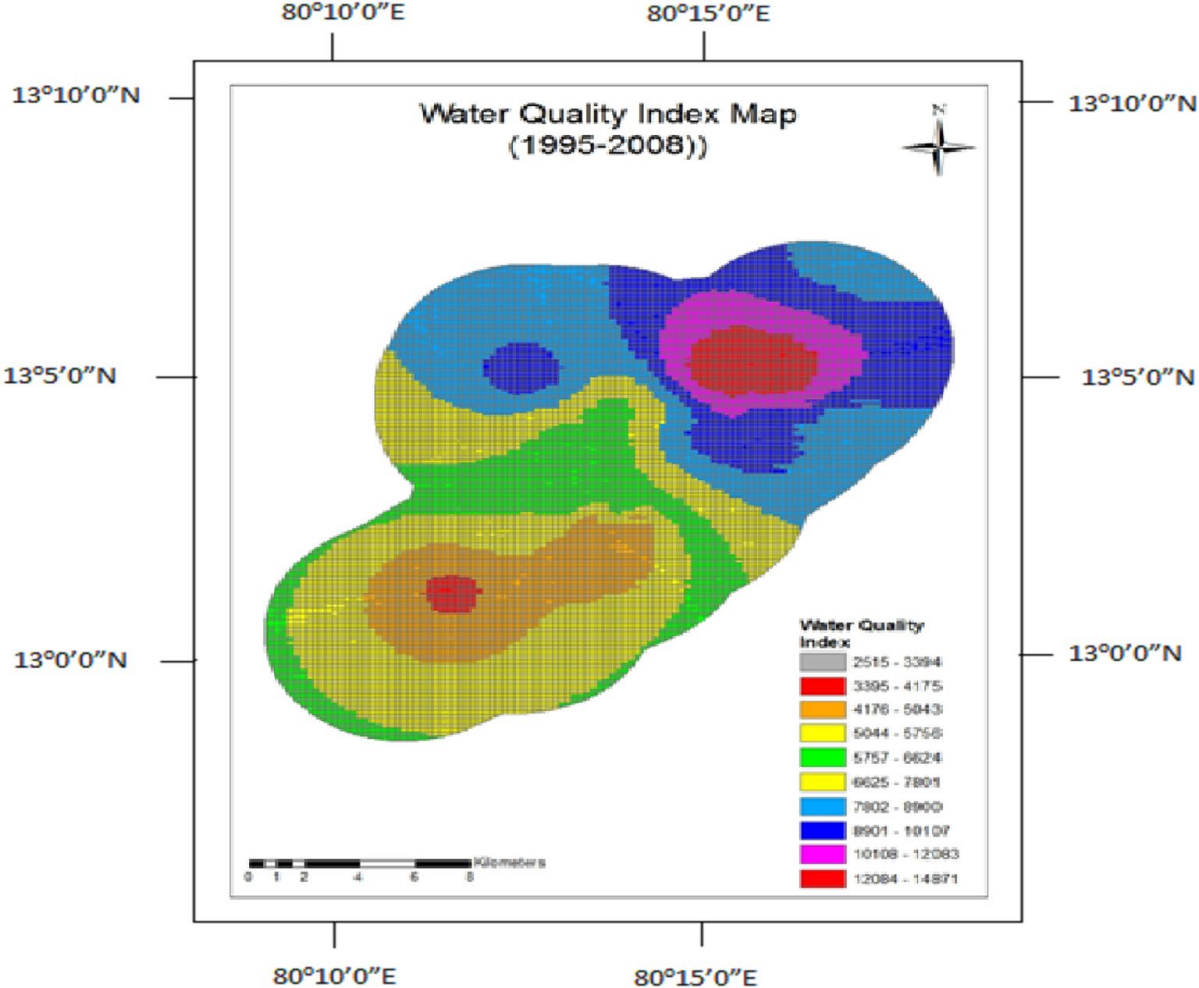
Water quality index map for 1995–2008.

**Figure 8:**
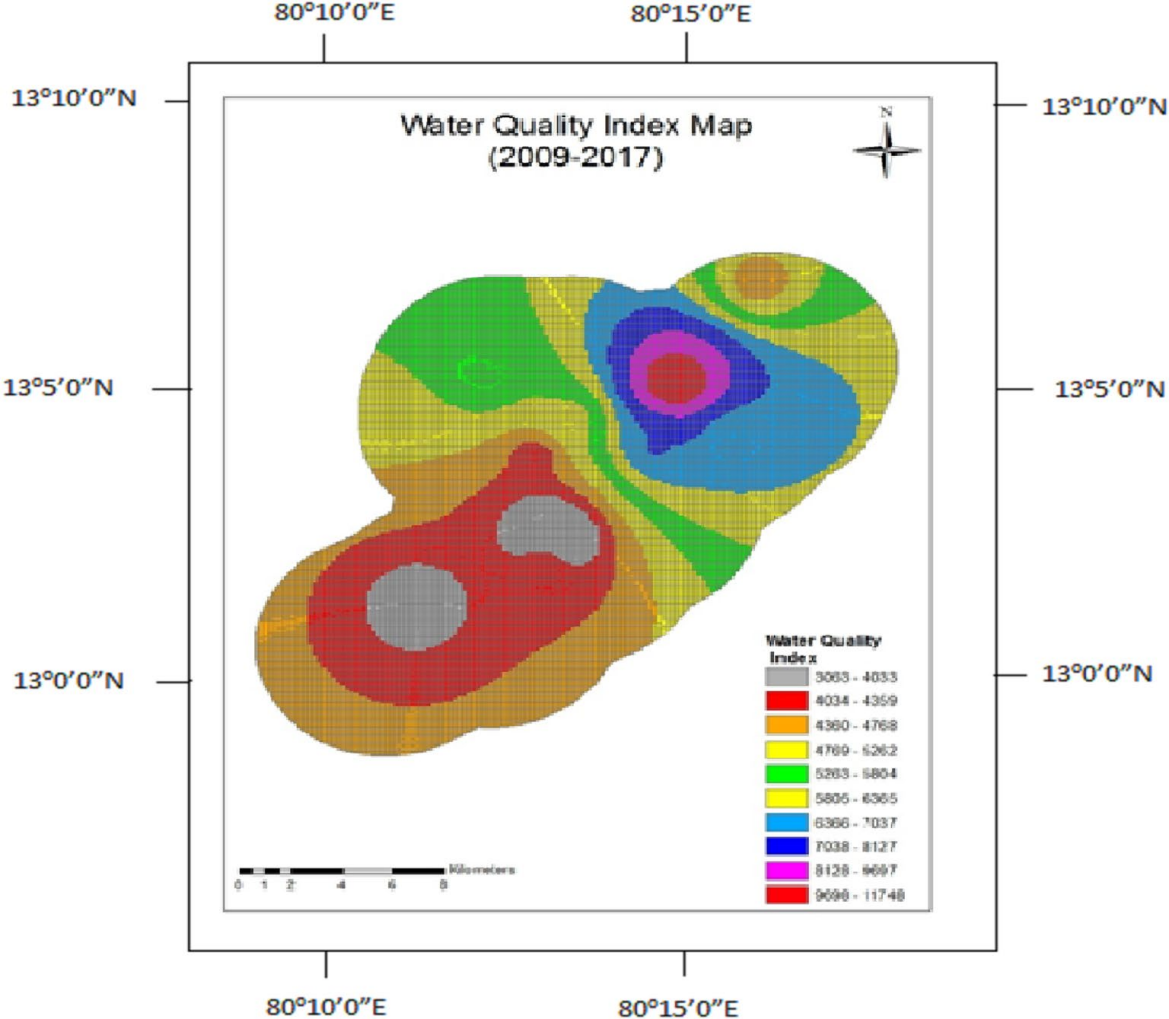
Water quality index map for 2009–2014.

Water Quality Index Maps (Figs. 7 and 8) clearly show that before the construction, the water quality got deteriorated over the North-East axis of the study area. After the construction, the higher values were seen in the Southwest axis in addition to the North-East axis. Hence the tunneling process makes a tremendous change in the concentration of the water quality parameters and also its mobilization.

### Temporal variation analysis using GIS

GIS software provides extensive functionality that allows a user to combine the spatial and attribute components of the data. This allows data to be queried and integrated into ways that no other approach can manage. The buffered study area was divided into polygons for the selected observation wells in the study area. Created polygons were extended to the extent of the buffered study area. The polygons got intersected with the buffered area.

The intersected polygon was subjected to query analysis. Queries included both Comparison and Boolean operators. The water quality parameters were paired, and the query was made with respect to the polygons created. They were selected on the basis of the conditions given in the query being satisfied. The analysis was carried out for the paired parameters like Total Dissolved-Total Hardness, pH-Electrical Conductivity, Total alkalinity-Chloride Calcium -Magnesium and Fluoride–Sulphate.

### pH and electrical conductivity

The concentration of hydrogen ions in water is measured by pH. pH greater than 7 shows alkaline nature, which is not fit for drinking. An electrical conductivity concentration of more than the permissible limit indicates the presence of dissolved inorganic substances in ionized form. The grouting process in tunneling influences the migration of these ionic species into the fissures, likely to increase the electrical conductivity^42^.

**Figure 9:**
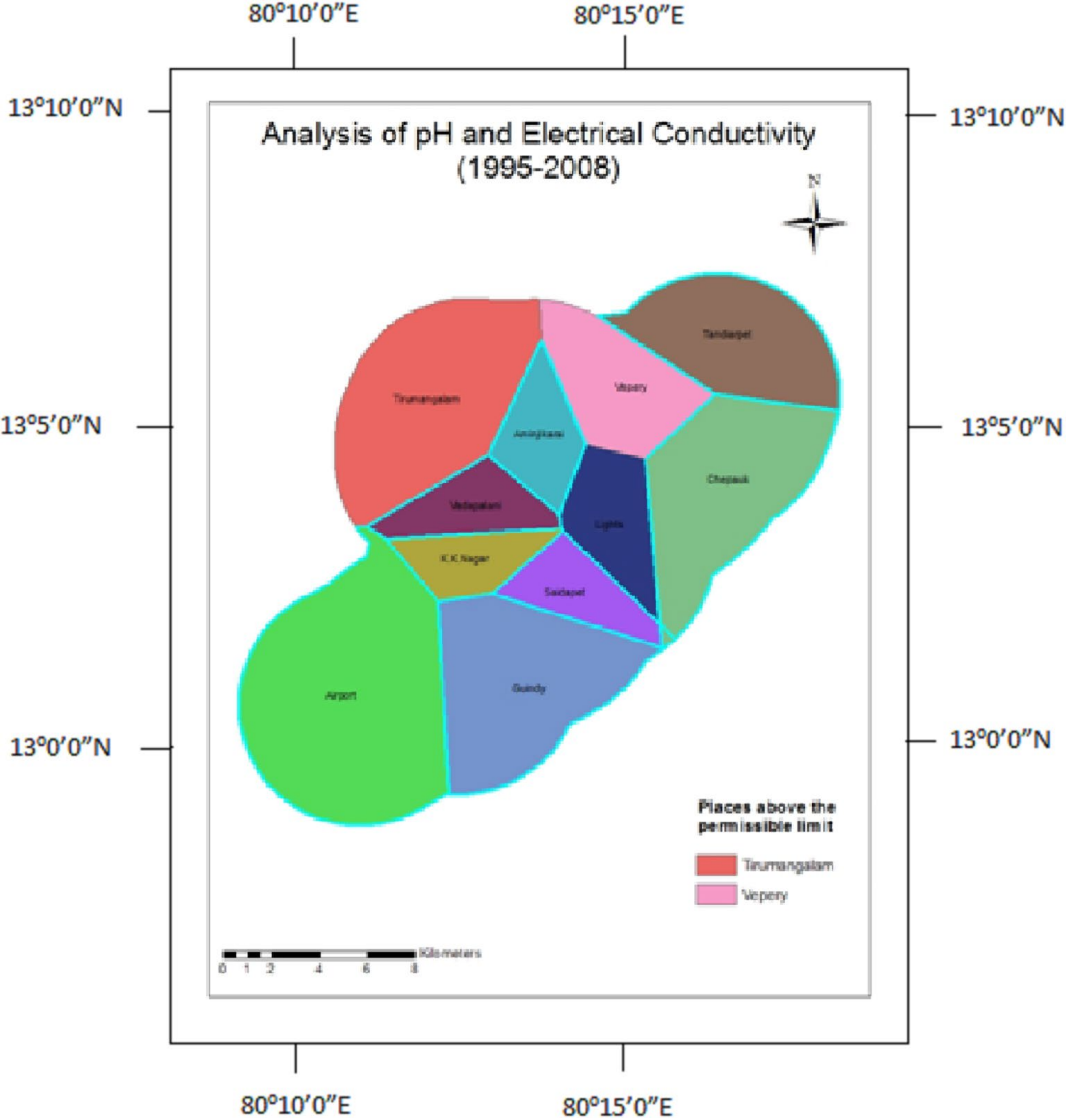
Analysis of pH and electrical conductivity for 1995–2008.

**Figure 10:**
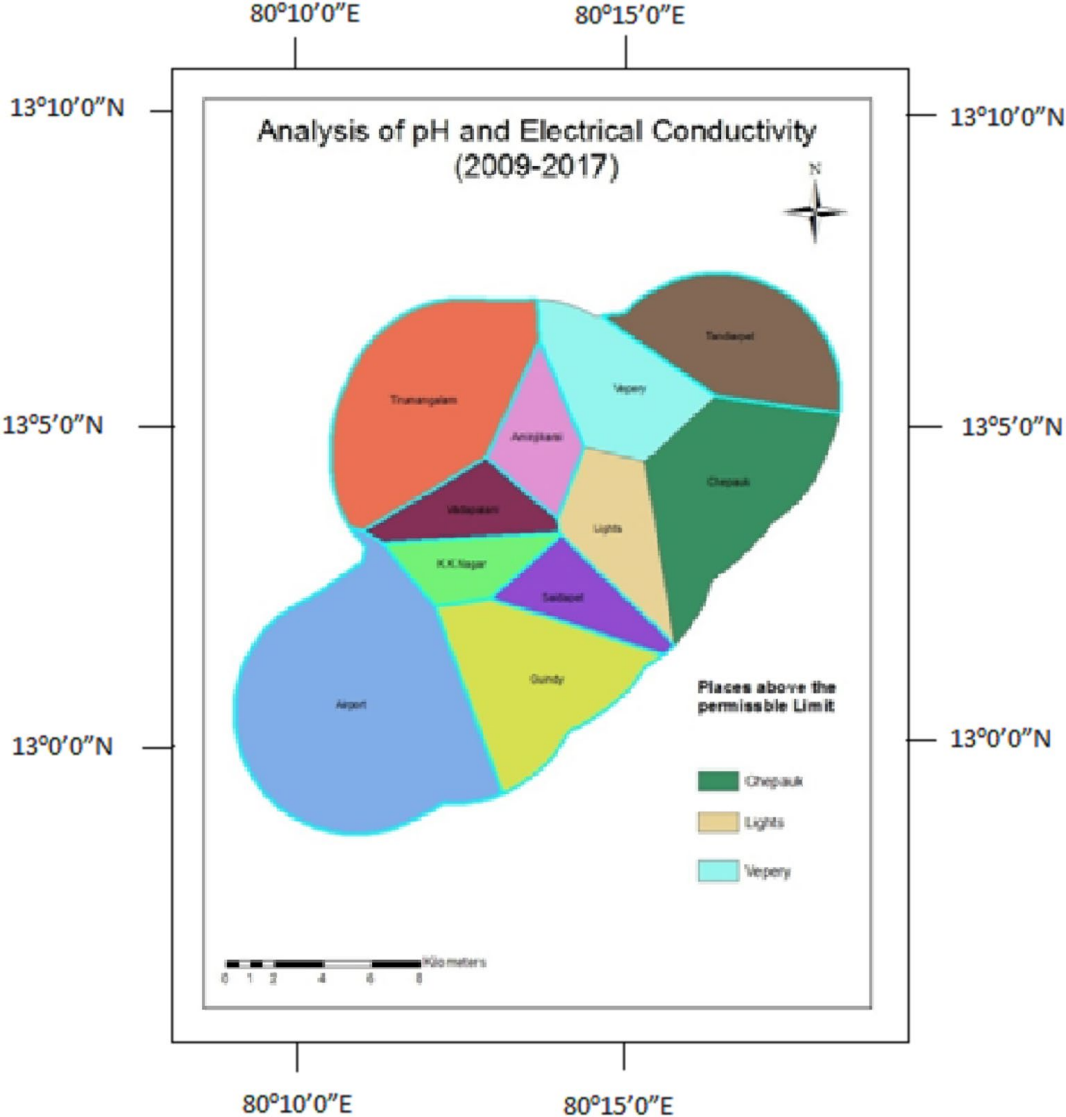
Analysis of pH and electrical conductivity for 2009–2014.

The analysis of pH and electrical conductivity shows higher values in the observation wells of Tirumangalam and Vepery before the Construction (Fig. 9). After the construction, the values were shown within the permissible limit in the observation wells of Chepauk, Thousand Lights, and Vepery (Fig. 10).

### Total dissolved solids and total hardness

**Figure 11:**
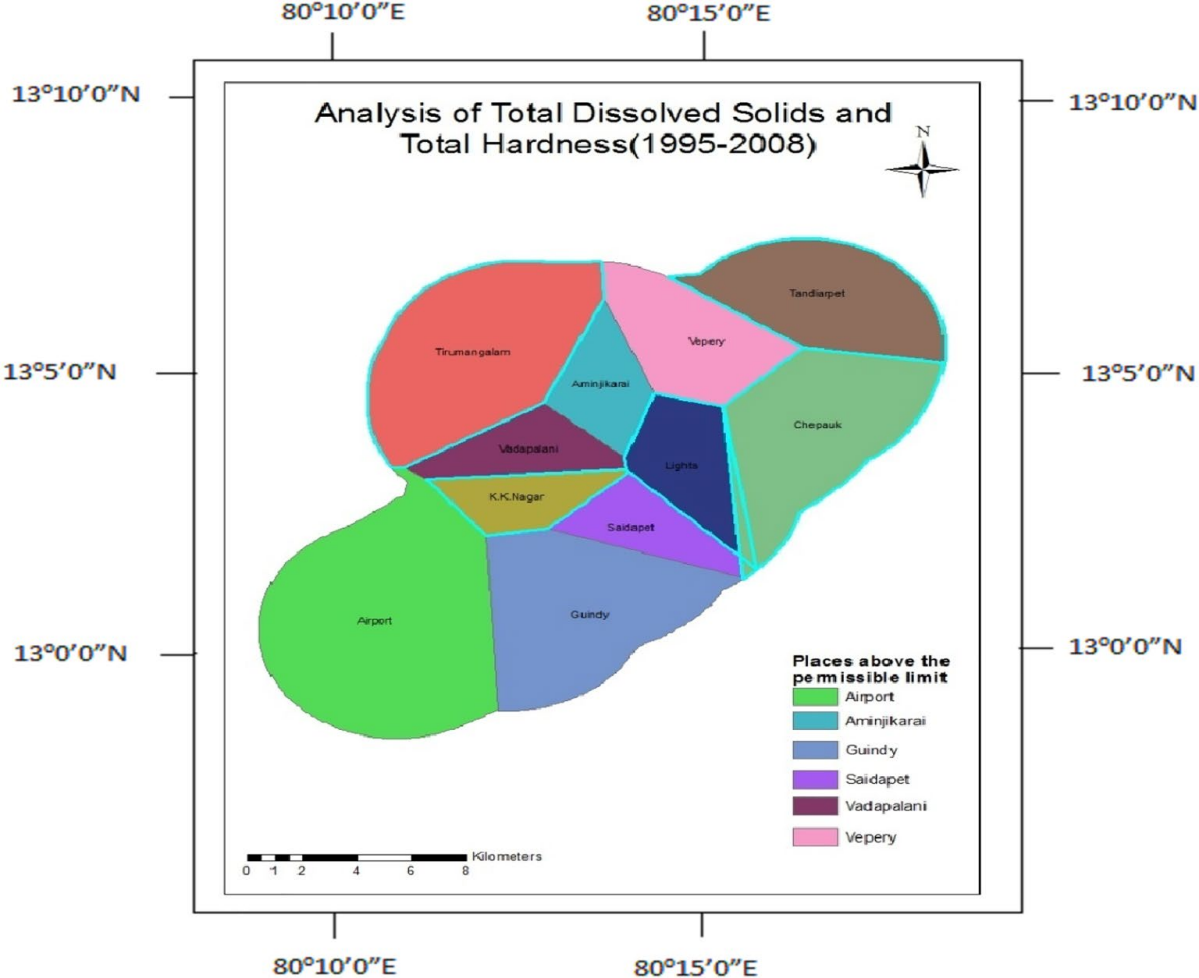
Analysis of total dissolved solids and total hardness for 1995–2008.

**Figure 12:**
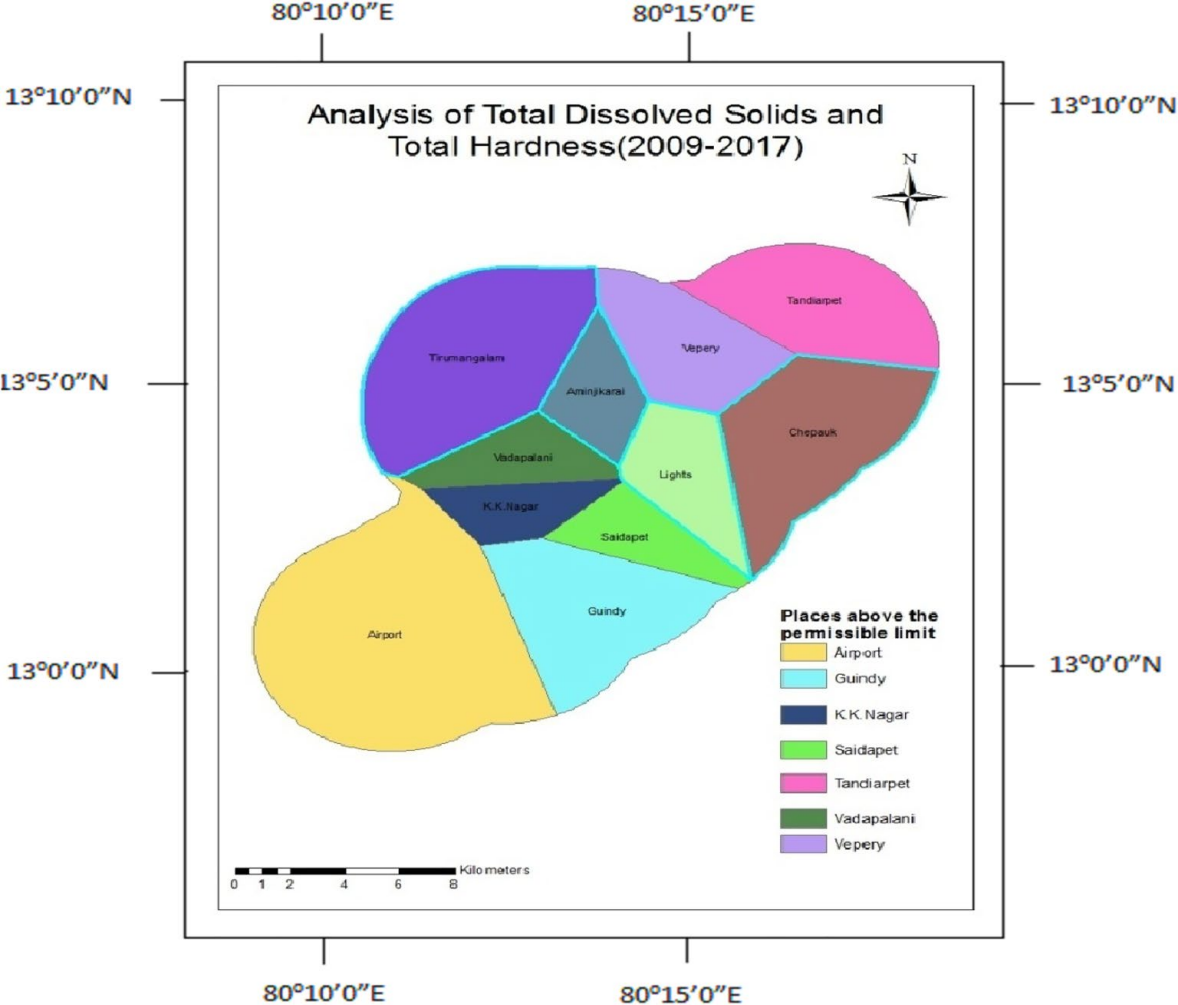
Analysis of total hardness and total dissolved solids for 2009–2017.

Total dissolved solids indicate the presence of a suspended form of organic and inorganic substances present in water. Total hardness occurs due to the presence of Calcium and Magnesium bicarbonates in the water. Total dissolved solids and total hardness parameters were found to be above the permissible limit in 50% of wells except for Tirumanglam, Tondiarpet, K.K.Nagar, Thousand Lights, and Chepauk (Fig. 11), but after the construction, 60% of wells got affected except Tirumangalam, Aminjikarai, Chepauk, and Thousand Lights (Fig. 12).

### Calcium and magnesium

**Figure 13:**
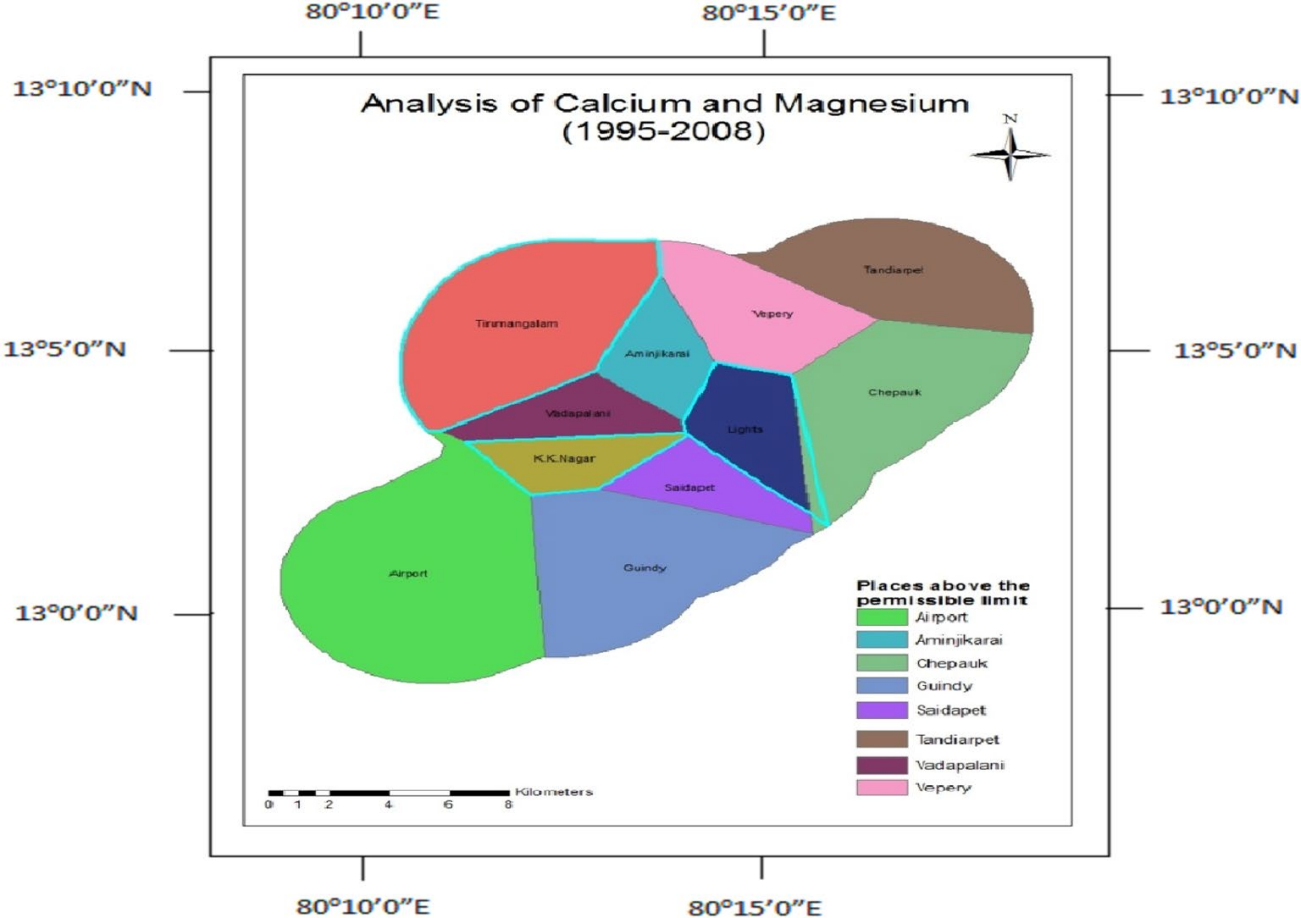
Analysis of calcium and magnesium for 1995-.

**Figure 14:**
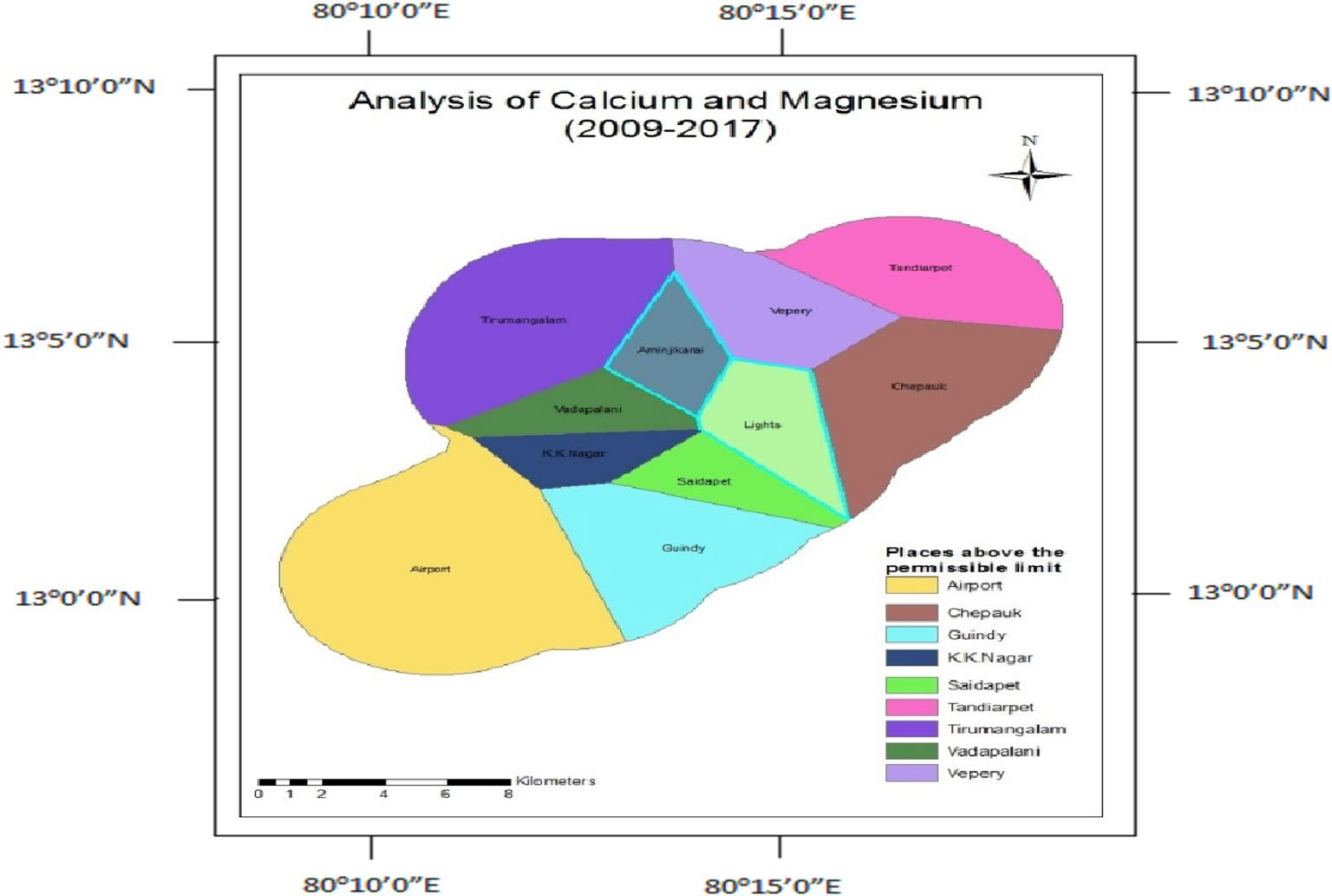
Analysis of calcium and magnesium for 2009–2014.

Calcium and Magnesium ions in water are related to hardness. The concentrations in excess in drinking water are unfit for drinking. Before the construction, Calcium and Magnesium concentrations were found to be within the limit in the observation wells like Tirumangalam, K.K.Nagar, and Thousand Lights (Fig. 13). After the construction, only Aminjikarai and Thousand Lights were within the permissible limit (Fig. 14).

### Total alkalinity and chloride

**Figure 15:**
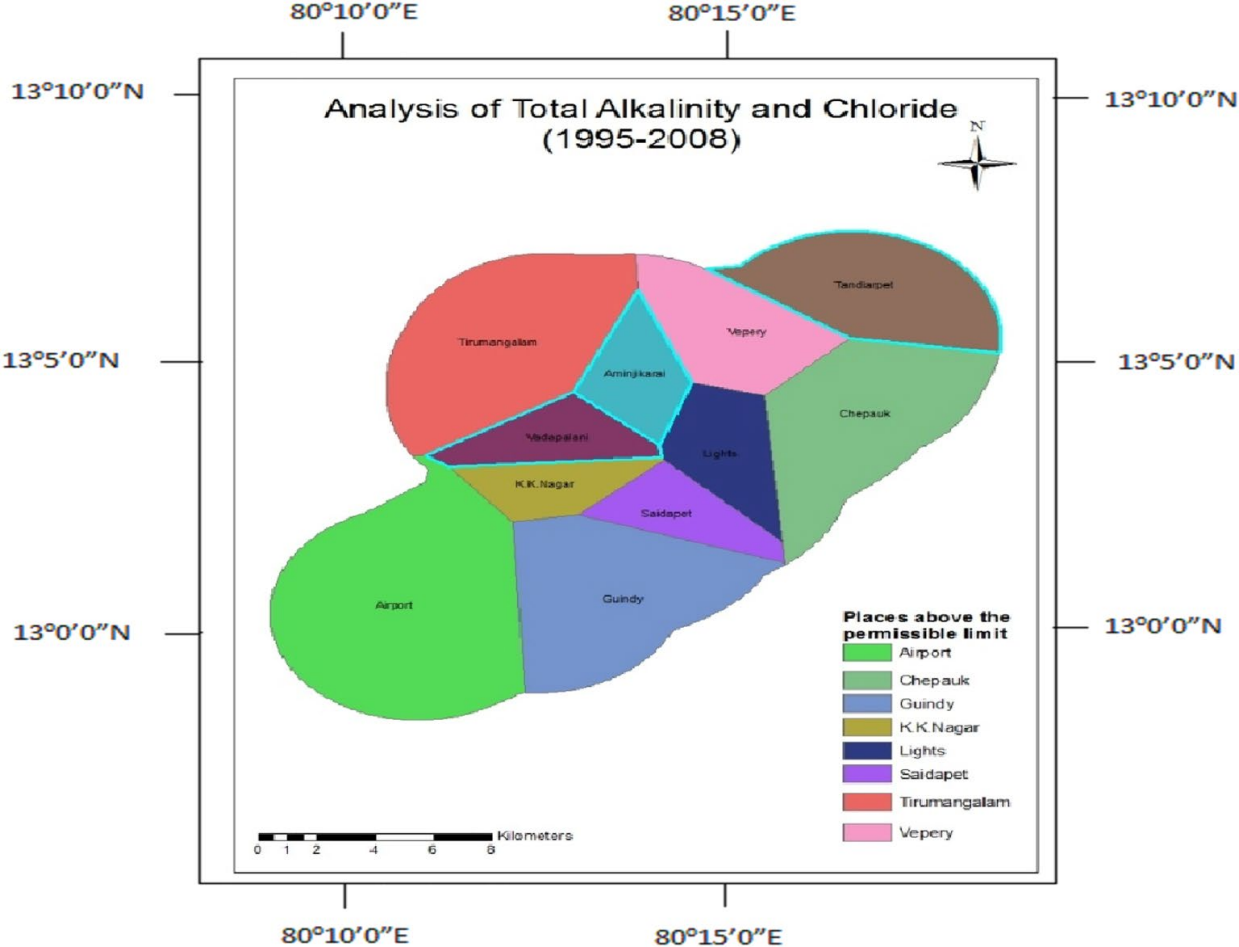
Analysis of total alkalinity and chloride for 1995–2008.

**Figure 16:**
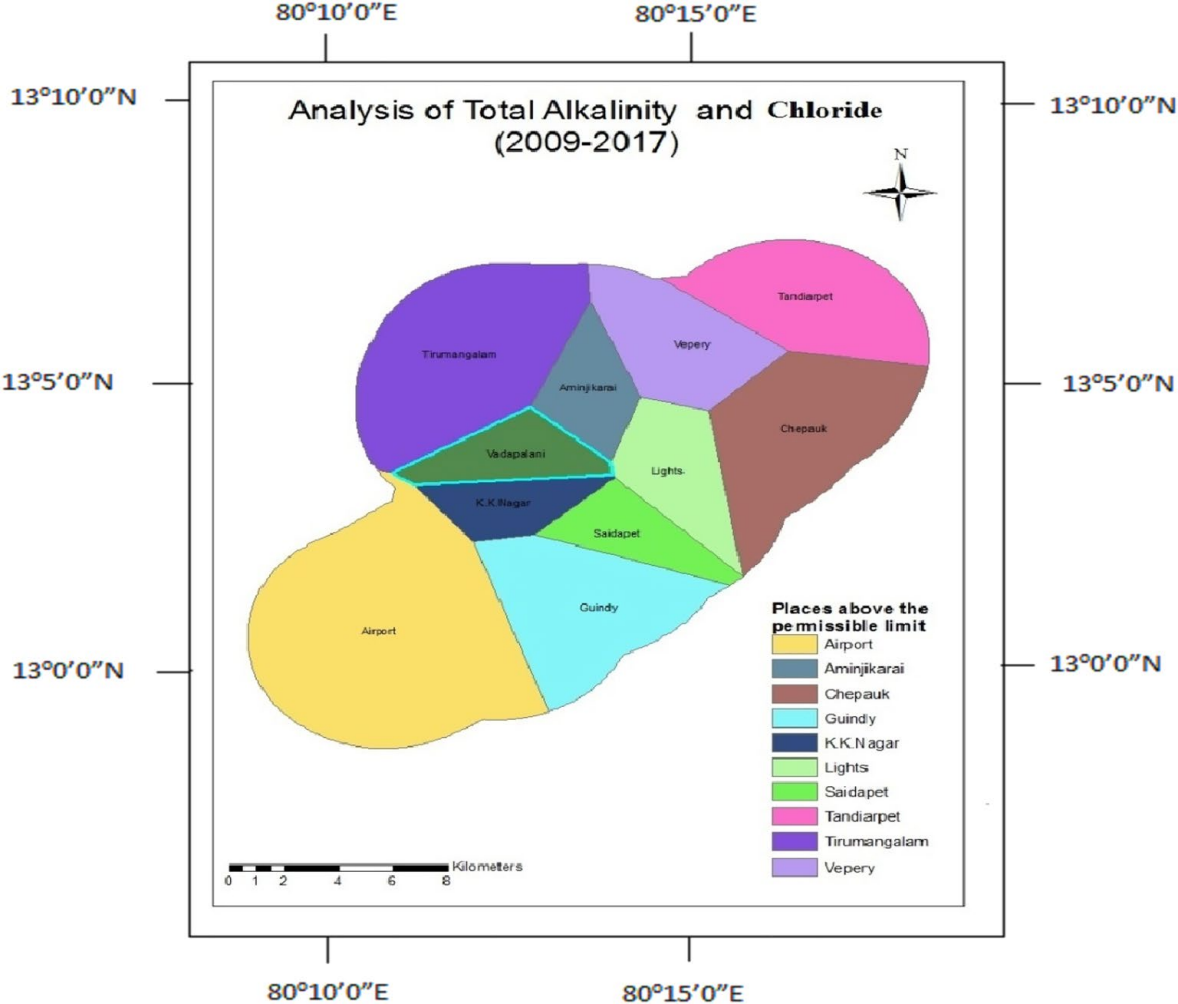
Analysis of total alkalinity and chloride for 2009–2014.

The presence of ions determines the alkalinity in water. The chloride content in water indicates its corrosive nature. The lack of an underground drainage system increases the chloride content of water. Analysis of total alkalinity and chloride shows only in Vadapalani well; the parameters are within the range after the construction (Fig. 15); before the construction, the wells like Tirumangalam, K.K.Nagar, and Thousand Lights are found to be good with the concentration of total alkalinity and chloride (Fig. 16).

### Fluoride and sulphate

**Figure 17:**
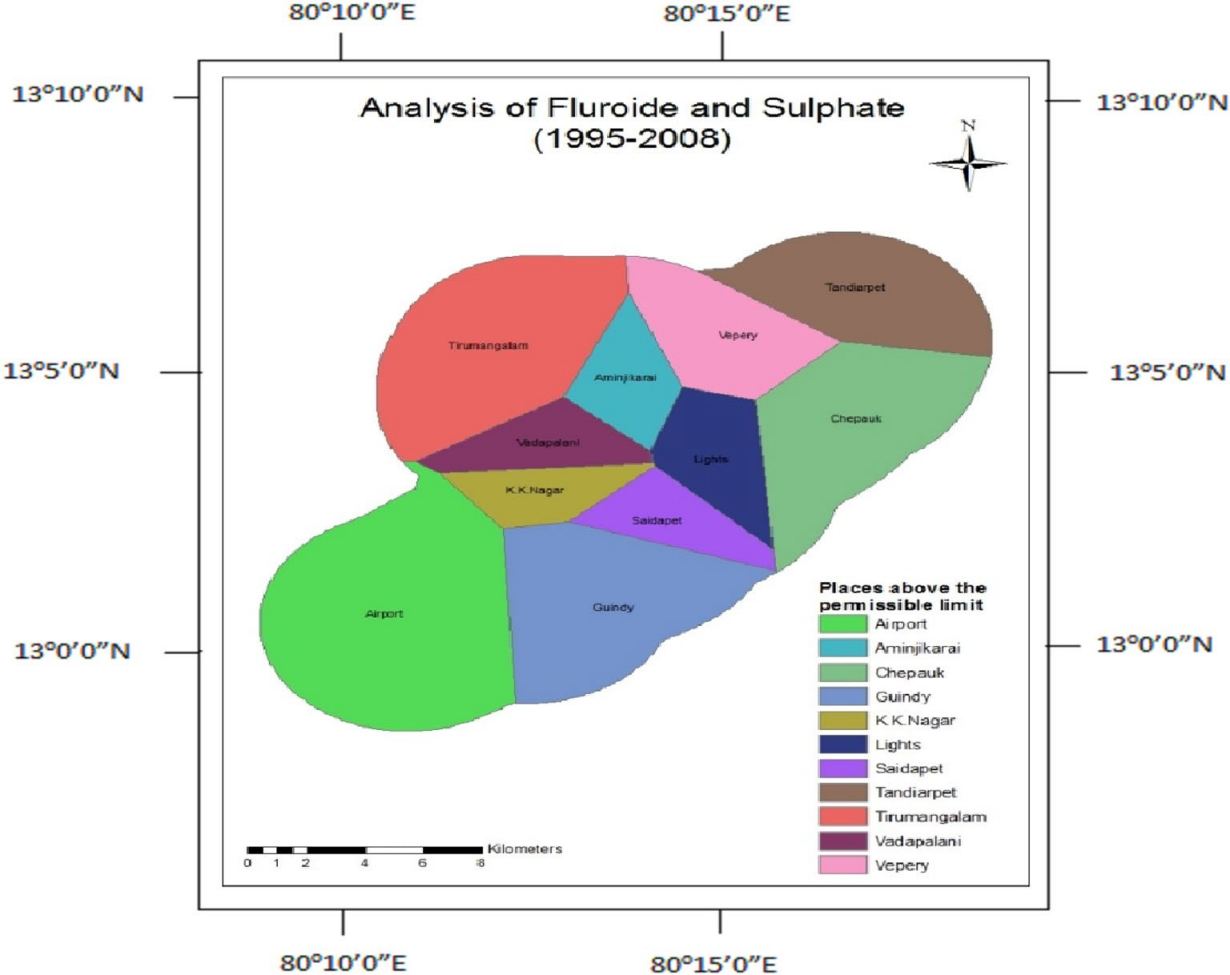
Analysis of fluoride and sulphate for 1995–2008.

**Figure 18:**
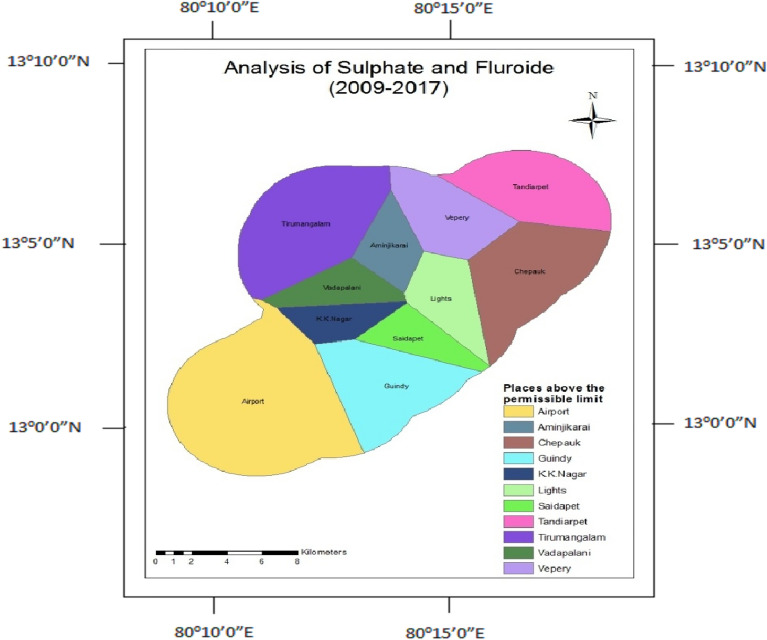
Analysis of fluoride and sulphate for 2009–2014.

Fluoride and Sulphate occur naturally in groundwater due to the breakdown of rocks and soils. Tunnelling involves breaking down the earth's crust. Hence, the concentration of fluoride and sulphate increased, and also the usage of cement, binders composed of gypsum (CaSO_4_), and other minerals leaching also increased the concentrations of Sulphate and Fluoride. Fluoride and Sulphate concentrations were found to be higher in both phases (Figs. 17 and 18).

## Conclusions

In the GIS environment, the water quality status in most of the observation wells is found to be improved by the usage of ferritin iron storage protein. pH and Electrical Conductivity were found to be got reduced and just above the permissible in the wells of Chepauk and Thousand Lights. 20% reduction in the total hardness and total dissolved solids in the wells of Tirumangalam, Aminjikarai, and Chepauk. Total alkalinity and chloride were found to show a good reduction in the pollutant level of Tirumangalam, K.K.Nagar, and Thousand Lights. Fluoride, sulphate, and calcium were found to be within the permissible limits after the usage of the nanoparticles.

The ferritin family protein reduces the water pollutant level of various water quality parameters. The pollutant level of pH is considerably reduced up to 0.9 because of the reduction of alkali salts as it gets absorbed by the Nano photocatalyst. The Electrical conductivity values show a considerable decrease from the earlier phase of the Metro Rail Corridor, about 200 mg/l. The total dissolved solids are connected to electrical conductivity; this pollutant also showed a considerable decrease to the amount of 110 mg/l. Calcium concentration, an average of 15 mg/l, is reduced from the earlier phase of construction. Total alkalinity was tested in the laboratory, and the test results showed a nearly 120 mg/l decrease from the contaminated level of water. The chloride levels of water in the construction of the foundation create more problems in the adsorption to the structure and deteriorate the structure very much. The ferritin reduces the chloride salts to an average of 75 mg/l from the earlier phase of construction. The fluoride salt occurs in nature in the tremendous occurrence is activated due to the construction of the foundation very deeply, but the ferritin molecules reduce up to the level of 0.2 mg/l, and the sulphate level of 50 mg/l decreases from the earlier phase of construction.

Water Quality Index values calculated for the two phases clearly show that the water quality around the Metro Rail Corridor decreased after the construction of Metro Rail. The Index mapping of the water shows the comparative difference in the concentrations after the construction of Metro Rail Corridors. The temporal variation clearly shows that the affected areas are more in the later phase when compared to the previous one. Also, due to the tunnelling effect, some parameters have reclaimed and reached their permissible limit.

The questionnaire survey of the community-dwelling on either of the corridors was taken. The output of the questionnaire survey was overlaid with the results obtained from the water quality status of the study area. About 70% of the respondents’ views matched the results obtained.

## Data Availability

The data used to support the findings of this study are included within the article.

## References

[1] Theil EC (1987). Ferritin: Structure, gene regulation, and cellular function in animals, plants, and microorganisms. Annu. Rev. Biochem..

[2] Srivastav AL, Kumar A (2021). An endeavor to achieve sustainable development goals through floral waste management: A short review. J. Clean. Prod..

[3] Watlington, K. Emerging nanotechnologies for site remediation and wastewater treatment. (Environmental Protection Agency Washington DC, 2005).

[4] Grevatt, P. US Environmental Protection Agency, Toxicological review of hexavalent chromium. Natl. Cent. Environ. Assessments, Off. Res. Dev. Washington, DC (1998).

[5] Raghavendra NS, Deka PC (2015). Sustainable development and management of groundwater resources in mining affected areas: a review. Procedia Earth Planet. Sci..

[6] Mohanavelu A (2020). Trends and Non-stationarity in groundwater level changes in rapidly developing Indian cities. Water.

[7] Khazaei E, Mackay R, Warner JW (2004). The effects of urbanization on groundwater quantity and quality in the Zahedan aquifer, southeast Iran. Water Int..

[8] Qun, M., Ying, G., Zhiqiang, L. & Xiaohui, T. Application of comprehensive water quality identification index in water quality assessment of river. In *2009 WRI Global Congress on Intelligent Systems* 1, 333–337 (2009).

[9] Luczaj J, Masarik K (2015). Groundwater quantity and quality issues in a water-rich region: Examples from Wisconsin, USA. Resources.

[10] Jat MK, Garg PK, Khare D (2008). Modelling of urban growth using spatial analysis techniques: a case study of Ajmer city (India). Int. J. Remote Sens..

[11] Loganathan D (2011). status of groundwater at Chennai city, India. Indian J. Sci. Technol..

[12] Saraf AK, Choudhury PR (1998). Integrated remote sensing and GIS for groundwater exploration and identification of artificial recharge sites. Int. J. Remote Sens..

[13] Han, M., Tian, X. & Xu, S. Research on data collection and database update of GIS based on GPS technology. In *Proceedings. 2005 IEEE International Geoscience and Remote Sensing Symposium*, 2005. IGARSS’05. vol. 2, 4 (2005).

[14] Kim, K.-S., Kim, M.-S. & Lee, K. On integrated scheme for vector/raster-based GIS’s utilization. in IGARSS’97. 1997 IEEE International Geoscience and Remote Sensing Symposium Proceedings. Remote Sensing-A Scientific Vision for Sustainable Development 1, 200–203 (1997).

[15] Gummadi S, Swarnalatha G, Venkataratnamma V, Vishnuvardhan Z (2014). Water quality index for groundwater of Bapatla Mandal, coastal Andhra Pradesh, India. Int. J. Environ. Sci..

[16] Deepika BV, Ramakrishnaiah CR, Naganna SR (2020). Spatial variability of ground water quality: A case study of Udupi district, Karnataka State, India. J. Earth Syst. Sci..

[17] Saravanan, R., Gracia, F. & Stephen, A. Basic principles, mechanism, and challenges of photocatalysis. In Nanocomposites for visible light-induced photocatalysis 19–40 (Springer, 2017).

[18] Gomes J, Lincho J, Domingues E, Quinta-Ferreira RM, Martins RC (2019). N-TiO2 photocatalysts: A review of their characteristics and capacity for emerging contaminants removal. Water.

[19] Ong CB, Ng LY, Mohammad AW (2018). A review of ZnO nanoparticles as solar photocatalysts: Synthesis, mechanisms and applications. Renew. Sustain. Energy Rev..

[20] Gómez-Pastora J (2017). Review and perspectives on the use of magnetic nanophotocatalysts (MNPCs) in water treatment. Chem. Eng. J..

[21] Umar K (2015). Synthesis, characterization of Mo and Mn doped ZnO and their photocatalytic activity for the decolorization of two different chromophoric dyes. Appl. Catal. A Gen..

[22] Loeb, S. K. *et al*. The technology horizon for photocatalytic water treatment: sunrise or sunset? (2018). 10.1021/acs.est.8b05041 30576114

[23] Graça CAL, Mendes MA, Teixeira ACSC, de Velosa AC (2020). Anoxic degradation of chlorpyrifos by zerovalent monometallic and bimetallic particles in solution. Chemosphere.

[24] Sadegh H (2017). The role of nanomaterials as effective adsorbents and their applications in wastewater treatment. J. Nanostruct. Chem..

[25] Qu X, Alvarez PJJ, Li Q (2013). Applications of nanotechnology in water and wastewater treatment. Water Res..

[26] Raliya R, Avery C, Chakrabarti S, Biswas P (2017). Photocatalytic degradation of methyl orange dye by pristine titanium dioxide, zinc oxide, and graphene oxide nanostructures and their composites under visible light irradiation. Appl. Nanosci..

[27] Liang X (2019). Removal effect on stormwater runoff pollution of porous concrete treated with nanometer titanium dioxide. Transp. Res. Part D Transp. Environ..

[28] Bhatia D, Sharma NR, Singh J, Kanwar RS (2017). Biological methods for textile dye removal from wastewater: A review. Crit. Rev. Environ. Sci. Technol..

[29] Sherman, J. Nanoparticulate titanium dioxide coatings, and processes for the production and use thereof. (2003).

[30] Ali I, AlGhamdi K, Al-Wadaani FT (2019). Advances in iridium nano catalyst preparation, characterization and applications. J. Mol. Liq..

[31] Abel, S. et al. Application of titanium dioxide nanoparticles synthesized by sol-gel methods in wastewater treatment. *J. Nanomater.* **2021** (2021).

[32] Abel, S. *et al.* Green synthesis and characterizations of zinc oxide (ZnO) nanoparticles using aqueous leaf extracts of coffee (Coffea arabica) and its application in environmental toxicity reduction. *J. Nanomater.* **2021** (2021).

[33] Bulcha, B. *et al.* Synthesis of zinc oxide nanoparticles by hydrothermal methods and spectroscopic investigation of ultraviolet radiation protective properties. *J. Nanomater.* **2021** (2021).

[34] Markos, M., Saka, A., Jule, L. T., Nagaprasad, N. & Ramaswamy, K. Groundwater potential assessment using vertical electrical sounding and magnetic methods: A case of Adilo Catchment, South Nations, Nationalities and Peoples Regional Government, Ethiopia. Concepts Magn. Reson. Part A, Bridg. Educ. Res. 2021, (2021).

[35] Abel, S. *et al.* Investigating the influence of bath temperature on the chemical bath deposition of nanosynthesized lead selenide thin films for photovoltaic application. *J. Nanomater.* **2022** (2022).

[36] Ramaswamy K, Jule LT, Subramanian K, Seenivasan V (2022). Reduction of environmental chemicals, toxicity and particulate matter in wet scrubber device to achieve zero emissions. Sci. Rep..

[37] Shimelisa, B. *et al.* Preparation of hydrated lime quality for water treatment: to reduce silica concentration from hydrated lime up to standard specification. In *Presented at the Virtual International Conference on New Strategies in Water Treatment and Desalination* (WTD-2021) vol. 21, 23 (2021).

[38] Ramaswamy K, Jule LT, Subramanian K, Seenivasan V (2022). Investigation on Pollution Control Device (PCD) in iron foundry industry to reduce environmental chemicals. PLoS ONE.

[39] Li B, Zhang S, Du J, Sun Y (2022). State-of-the-art in cutting performance and surface integrity considering tool edge micro-geometry in metal cutting process. J. Manuf. Process..

[40] Malik A (2014). Electrical and optical properties of nickel-and molybdenum-doped titanium dioxide nanoparticle: improved performance in dye-sensitized solar cells. J. Mater. Eng. Perform..

[41] Lyu H-M, Shena S-L, Zhou A (2021). The development of IFN-SPA: A new risk assessment method of urban water quality and its application in Shanghai. J. Clean. Prod..

[42] Saka, A. *et al.* Polymeric droplets on SiO_2_ nanoparticles through wastewater treatment of carbon-based contaminants in photocatalytic degradation. *J. Nanomater.* **2022** (2022).

